# Ultrasound Findings in a Case of Myeloid Sarcoma of the Breast

**DOI:** 10.5334/jbr-btr.986

**Published:** 2016-02-02

**Authors:** Arzu Ozsoy, Betul Akdal Dolek, Nurdan Barca, Hafize Aktas, Levent Araz, Sezer Kulacoglu

**Affiliations:** 1 Department of Radiology, Ankara Numune Training and Research Hospital, Ankara, Turkey; 2 Department of Pathology, Ankara Numune Training and Research Hospital, Ankara, Turkey

**Keywords:** Myeloid sarcoma, breast, ultrasonography

## Abstract

Myeloid sarcoma is a rare, solid extramedullary tumor originating from immature granulocytic cells or monocytes. Breast involvement without an aleukemic or myeloproliferative disorder is very infrequent. A 21-year-old female patient was admitted with bilateral palpable breast masses for four months. The patient had given birth approximately one year ago. The ultrasonographic examination revealed multiple, oval shaped—some of them with microlubulated margins—hypoechoic, solid masses of which, the largest mass measured 4.5 × 2.5 cm, evaluated as BI-RADS 4. The histopthological examination suggested hematolymphoid neoplasm. In the differential diagnosis of solid breast lesions, myeloid sarcoma should be kept in mind even without hematological findings. Early diagnosis of this tumor is important for the effectiveness of the medical treatment.

## Introduction

Myeloid sarcoma, also known as extramedullary myeloid cell tumor, myeloblastoma or chloroma, is a rare, solid extramedullary tumor originating from immature granulocytic cells or monocytes. It frequently invades the bone, soft tissues, lymph nodes, skin, and paranasal sinuses [1,2,3]. Myeloid sarcoma, which can be seen in 2–14% of acute myeloid leukemia (AML) patients, is mostly seen together with chronic myeloid leukemia or myelodysplastic syndromes [1,2,3,4,5]. Breast involvement without an aleukemic or myeloproliferative disorder is extremely rare [3,4,6,7,8,9].

Although breast involvement is very rare in the literature, a limited number of cases showing bilateral breast involvement diagnosed during imaging have been reported [3,6,7,8,9].

In this report, we present a patient who was admitted with bilateral breast masses, diagnosed as myeloid sarcoma and referred to the Medical Oncology Department for further treatment.

## Case Report

A 21-year-old female patient was admitted with bilateral painless palpable breast masses for four months, which had increased in size over that period. The patient had given birth approximately one year before, but her medical and family histories were otherwise unremarkable.

**Figure 1:**
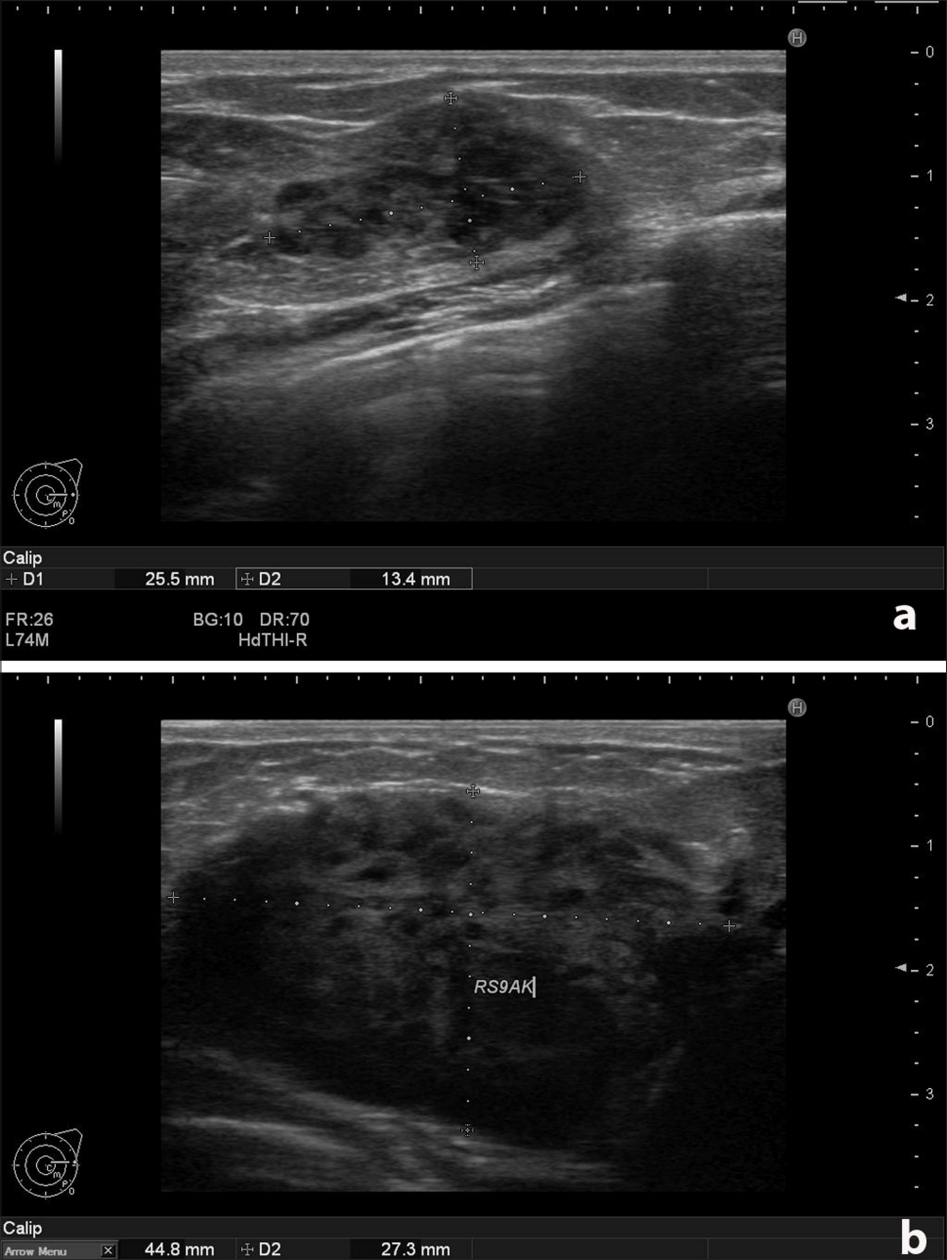
**(a)** Left breast. Solid, circumscribed mass. **(b)** Right breast. The largest solid mass measured 4.5 × 2.7 cm.

**Figure 2:**
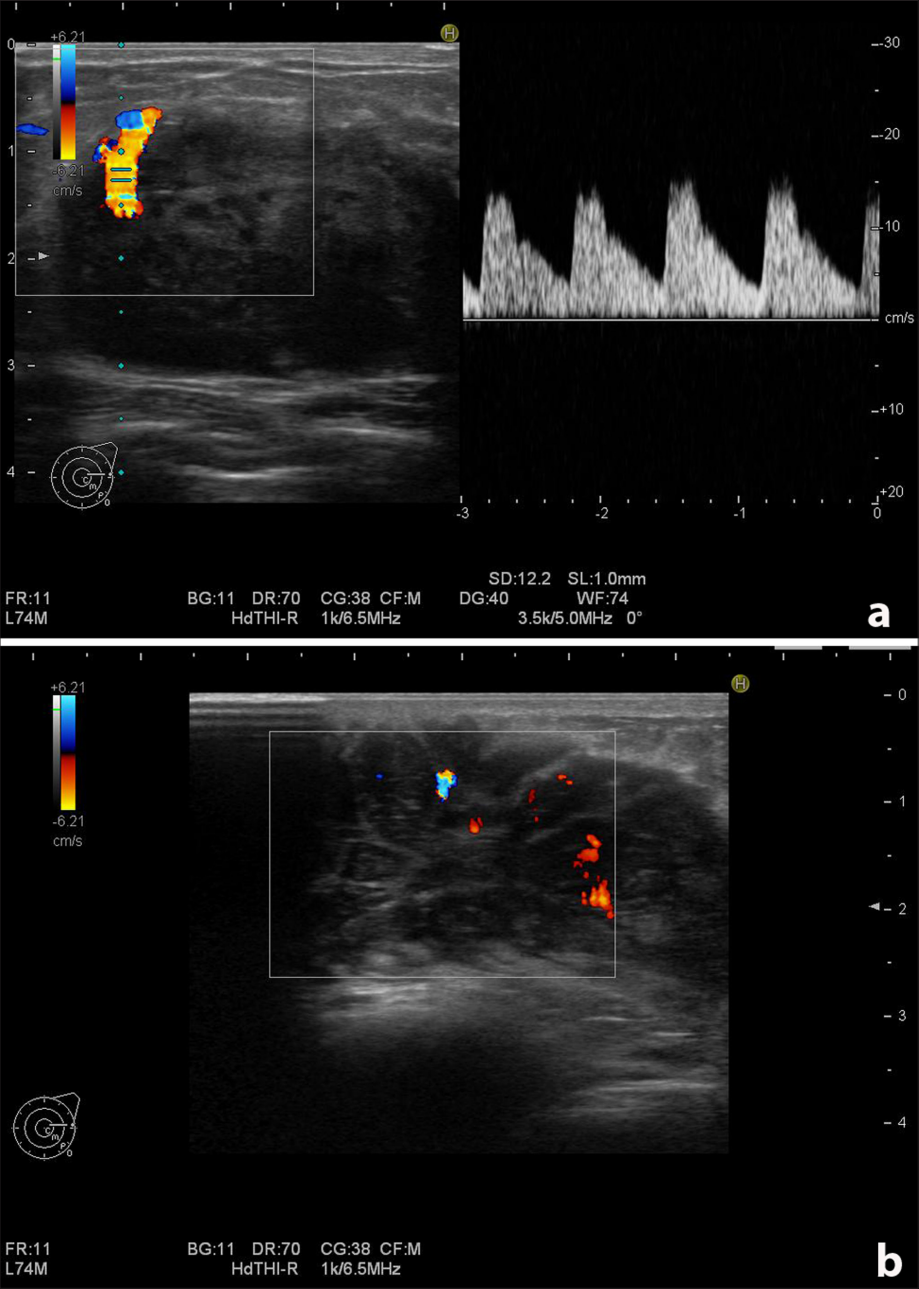
Doppler examination of the largest mass on the right breast. **(a)** Increased vascularity at the contours of the mass. **(b)** Increased vascularity in the internal part of the mass.

On physical examination, there were multiple palpable mass lesions in the lower quadrant in both breasts, with the largest being 4.5 cm in diameter in the right breast. There were no palpable axillary mass and no breast discharge or retraction of the areola. The ultrasonographic examination (Hitachi-Preius, 13–8 MHz) revealed multiple lobulated, hypoechoic, solid masses with circumscribed contours (Figure 1a). The largest mass measured 4.5 × 2.7 cm (Figure 1b). Power Doppler examination demonstrated increased vascularity in the masses (Figure 2a and b).

Since the patient was 21-years-old and without family risk, no further diagnostic modalities, such as mammography or MRI, was recommended.

**Figure 3:**
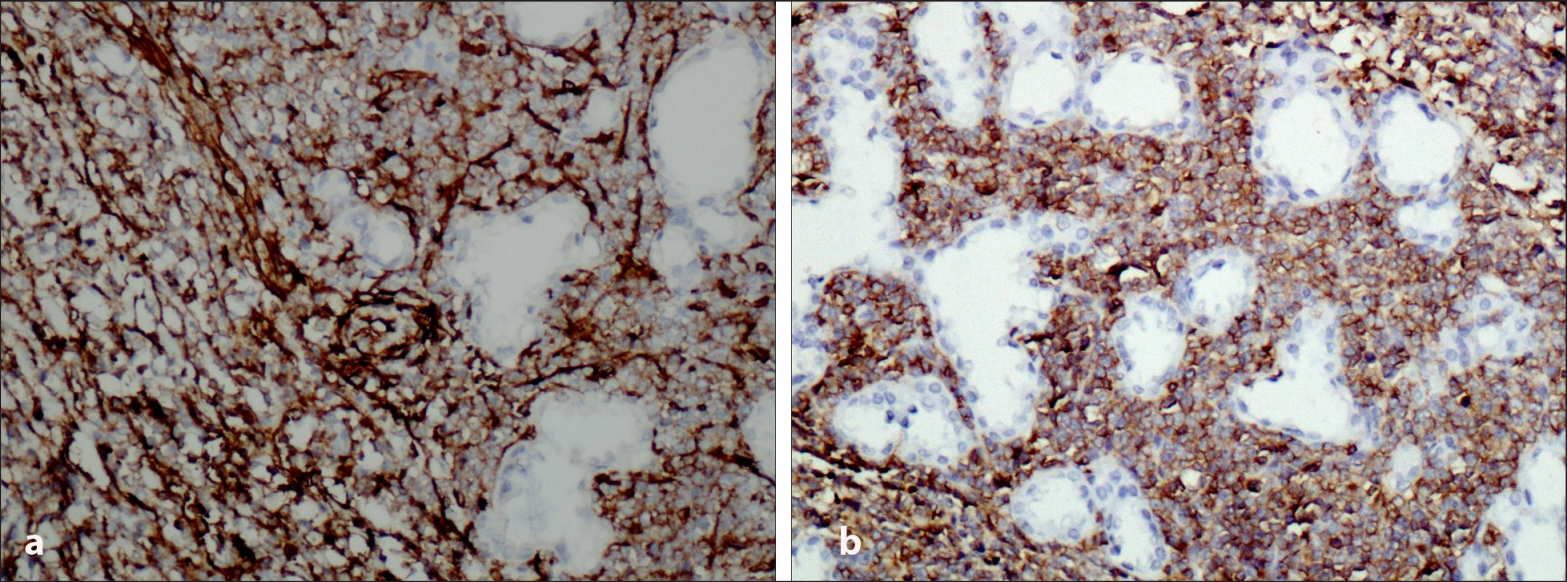
Histopathologic examination. **(a)** Strong positivity for CD34, ×200 (Figure 3a) and (b) CD43, ×200.

These findings were evaluated as BI-RADS 4, and biopsy was recommended. Core biopsy was non-diagnostic. After excisional biopsy, microscopic examination revealed infiltrating uniform, monotonous tumor cells with scanty cytoplasm between the breast lobules, showing lactational changes. These morphologic features suggested a hematolymphoid neoplasm. A broad immunohistochemical study was performed for confirmation and subtyping, and the results were as follows: The tumor cells showed strong positivity for CD34, CD43, CD99, Tdt, and bcl-2 protein (Figure 3a and b). CD79a and CD117 were weak-to-moderate positive in most cells, whereas some cells were strongly positive. There was focal positivity with myeloperoxidase. With CD68, there was strong positivity in some cells and faint positivity in others.

Peripheral smear and bone marrow examination were performed after the diagnosis of myeloid sarcoma, but revealed normal findings.

In the laboratory examination, the only pathological finding was a mild elevation in CA19-9 levels. In the whole body radiological examination, there were no other organ pathologies. The patient was diagnosed with myeloid sarcoma and chemotherapy was started. High dose cytarabine and idarubicine regime was used. This resulted in nearly total disappearance of the masses in both breasts. During two years follow-up the patient was disease free.

## Discussion

Myeloid sarcoma is a rare extramedullary hematological malignant tumor that originates from granulocytic precursor cells. It was first described by the English clinician A. Burns in 1811 [10]. Dock and Warthin [11] reported its relation with acute leukemia in 1902.

Breast involvement is rare [2]. There are only a limited number of cases reported in the literature with a diagnosis of myeloid sarcoma with breast involvement without AML history. Shera et al. made a literature review and revealed 19 cases of myeloid sarcoma without AML history until 2001 [8]. Similarly Thachil et al. could identify only 13 cases of myeloid sarcoma of the breast [9]. A recent study revealed 12 cases of myeloid sarcoma without AML history [6]. Moreover, in a population based analysis, Movassaghian et al. reported that between 1973 and 2010, 49.039 AML patients were identified. In the same period there were only four cases of breast myeloid sarcoma without AML [3].

Bilateral breast involvement without AML is extremely rare. Shera et al. reported two bilateral cases out of 19 myeloid sarcomas [8]. Thachil et al. made a literature search between 1970 and 2006, and could identify only four bilateral cases out of 13 myeloid sarcomas [9]. Similarly Nalwa reported two bilateral cases out of 12 myeloid sarcomas of the breast. [6] The age at presentation of isolated myeloid sarcoma cases ranges from 16–73 years. [8,9]. Patients usually present with painless breast masses [6,7]. Similar to the findings in the literature, our case was a 21-year-old female with the sole finding of bilateral palpable breast masses.

Ultrasonographic findings of myeloid sarcoma are variable. Thachil et al. reported that breast myeloid sarcoma shows homogenous areas of low attenuation, with well-or-ill-defined margins [9]. Guermazi et al. [12] reported that breast masses in myeloid sarcoma were irregularly shaped, heterogeneous, hypoechoic masses with visible posterior acoustic shadows. In our case, the mass lesions were visualized as hypoechoic, solid masses with oval shaped, circumscribed contours. Beacuse of their rarity and variable sonographic findings these lesions are difficult to differentiate from benign lesions (such as fibroadenoma, benign phyllodes tumor, granolmatous mastitis) or metastatic or hematologic malignancies. Therefore myeloid sarcoma should always be kept in mind in the differential diagnosis of such lesions. The prognosis of isolated aleukemic breast myeloid sarcoma cases is better than lesions with AML [3]. Studies indicated that, if left untreated, isolated myeloid sarcoma evolves into AML within one year [5,13]. Therefore early diagnosis and treatment is important. There is no consensus in the optimal treatment of the myeloid sarcoma [3]. However AML type systemic chemoteraphy is the most frequently used treatment protocol [6,8]. Our patient received a similar AML type chemoteraphy regime in which resulted nearly in total disappearance of the masses in both breasts.

## Conclusion

The appearance of myeloid sarcoma in the absence of leukemia or myeloproliferative disorders is rare. In the differential diagnosis of solid breast lesions on ultrasound, myeloid sarcoma should be kept in mind even without hematological findings. Early diagnosis of this tumor is important for the effectiveness of the treatment and patient survival.

## Competing Interests

The authors declare that they have no competing interests.

## References

[1] Kim SJ, Hong WS, Jun SH, Jeong SH, Kang SY, Kim TH (2013). Granulocytic sarcoma in breast after bone marrow transplantation. J Breast Cancer.

[2] Delporte F, Voorhoopf LJ, Lodewyck T, De Paepe P (2011). Primary granulocytic sarcoma of the breast: a case report and review of the literature. Eur J Gynaecol Oncol.

[3] Movassaghian M, Brunner AM, Blonquist TM (2014). Presentation and outcomes among patients with isolated myeloid sarcoma: a surveillance, epidemiology, and end results database analysis. Leuk Lymphoma.

[4] Ngu IW, Sinclair EC, Greenaway S, Greenberg ML (2001). Unusual presentation of granulocytic sarcoma in the breast: a case report and review of the literature. Diagn Cytopathol.

[5] Neiman RS, Barcos M, Berard C, Bonner H, Mann R, Rydell RE (1981). Granulocytic sarcoma: a clinicopathologic study of 61 biopsied cases. Cancer.

[6] Nalwa A, Nath D, Suri V, Jamaluddin MA, Srivastava A (2015). Myeloid sarcoma of the breast in an aleukemic patient: a rare entity in an uncommon location. Malays J Pathol.

[7] Valbuena JR, Admirand JH, Gualco G, Medeiros LJ (2005). Myeloid sarcoma involving the breast. Arch Pathol Lab Med.

[8] Shea B, Reddy V, Abbitt P, Benda R, Douglas V, Wingard J (2004). Granulocytic sarcoma (chloroma) of the breast: a diagnostic dilemma and review of the literature. Breast J.

[9] Thachil J, Richards RM, Copeland G (2007). Granulocytic sarcoma–a rare presentation of a breast lump. Ann R Coll Surg Engl.

[10] Burns A, Pattison GS (1811). Observations of Surgical Anatomy. Head and Neck.

[11] Dock G, Warthin AS (1904). A new case of chloroma with leukemia. Trans Assoc Am Phys.

[12] Guermazi A, Quoc SN, Socie G (2000). Myeloblastoma (chloroma) in leukemia: case 1. Granulocytic sarcoma (chloroma) of the breast. J Clin Oncol.

[13] Yamauchi K, Yasuda M (2002). Comparison in treatments of nonleukemic granulocytic sarcoma: report of two cases and a review of 72 cases in the literature. Cancer.

